# Complementarity among plant growth promoting traits in rhizospheric bacterial communities promotes plant growth

**DOI:** 10.1038/srep15500

**Published:** 2015-10-27

**Authors:** Mangal Singh, Ashutosh Awasthi, Sumit K. Soni, Rakshapal Singh, Rajesh K. Verma, Alok Kalra

**Affiliations:** 1 Department of Microbial Technology, CSIR- Central Institute of Medicinal and Aromatic Plants, P.O. CIMAP, Lucknow 226015, India

## Abstract

An assessment of roles of rhizospheric microbial diversity in plant growth is helpful in understanding plant-microbe interactions. Using random combinations of rhizospheric bacterial species at different richness levels, we analysed the contribution of species richness, compositions, interactions and identity on soil microbial respiration and plant biomass. We showed that bacterial inoculation in plant rhizosphere enhanced microbial respiration and plant biomass with complementary relationships among bacterial species. Plant growth was found to increase linearly with inoculation of rhizospheric bacterial communities with increasing levels of species or plant growth promoting trait diversity. However, inoculation of diverse bacterial communities having single plant growth promoting trait, i.e., nitrogen fixation could not enhance plant growth over inoculation of single bacteria. Our results indicate that bacterial diversity in rhizosphere affect ecosystem functioning through complementary relationship among plant growth promoting traits and may play significant roles in delivering microbial services to plants.

Microbial diversity in soil plays critical roles in ecosystem functioning including a large number of important processes like nutrient cycling^1^,^2^,^3^, promotion of plant growth and health^4^,^5^. These services are indispensable in agriculture and may enhance productivity in a sustainable manner. However, the functional role of microbial diversity in relation to plant productivity is poorly understood. Studying the functioning of rhizospheric microbial diversity for enhancing plant productivity will be helpful in understanding plant-microbe interactions. Diverse communities may use resources more efficiently and are more productive and stable under mild environmental fluctuations than monocultures^6^,^7^. Positive relationships between microbial diversity and ecosystem functioning have already been established where productivity, stability and resistance to biotic invasion increases at higher levels of diversity^8^,^9^,^10^,^11^. However, negative interactions in microbial communities are not uncommon^12^ and increasing frequency of antagonistic interactions at higher levels of species richness may result in the collapse of the whole community^13^.

Beneficial effects of biodiversity in microbial communities may enhance their survival and fitness and result in an overall increase in microbial biomass. Rhizospheric bacteria enhance plant productivity through various plant growth promoting activities such as nitrogen fixation, increasing the availability of essential nutrients, hormonal modulation and antagonising the growth of deleterious microbes^14^. Functional diversity is an important determinant of ecosystem processes^15^. The relationship between phylogenetic diversity and ecosystem functioning indicates that related organisms may share functional traits^16^,^17^. However, the distribution of functional traits in bacterial taxa is not fully understood and becomes complicated due to horizontal gene transfer and high evolutionary change^18^,^19^.

In a heterogeneous environment, biodiversity can increase ecosystem functioning through niche partitioning, habitat modification and direct facilitation to other organisms. For example, nitrogen fixers may provide extra nitrogen to other microbes, thus increasing their population, which, in turn, may facilitate nitrogen fixation through increasing availability of other essential nutrients like phosphorus. Here we study the functioning of microbial diversity in the rhizosphere and its role in plant growth. Bacterial strains were isolated from rhizospheric soil of *Ocimum sanctum* L. (Lamiaceae) on the basis of their common occurrence in the plant rhizosphere and their fast growing capability on nutrient agar medium. We hypothesized that the diversity in plant growth promoting traits in a bacterial community will be beneficial to plant growth, and functionally diverse bacterial community will promote more plant growth than simplified community.

## Materials and Methods

### Isolation and molecular characterization of rhizospheric bacteria

Seeds of *Ocimum sanctum* (cv. CIM-Angana) were obtained from the National Gene Bank for Medicinal and Aromatic Plants at CSIR-Central Institute of Medicinal and Aromatic plants (CSIR-CIMAP), Lucknow, India. Surface sterilized seeds were grown in organic farming experimental fields. Six weeks old healthy plants were uprooted and the soil firmly attached to the roots was collected after removing loosely adhered soil by shaking vigorously. One gram of homogenised soil sample was serially diluted in saline water (0.85%) and aliquot of 100 μL was spread on nutrient agar plates (peptic digest of animal tissue, 5 gL^−1^; beef extract, 1.5 gL^−1^; yeast extract, 1.5 gL^−1^; NaCl, 5 gL^−1^; agar 15 gL^−1^; HiMedia Laboratories India). After incubation for 2–7 days at 28 °C, the resulting conspicuous colonies were picked and purified by repeatedly streaking on nutrient agar. More than 50 bacterial isolates were purified and stored at −80 °C in 10% glycerol. Twenty-three bacterial strains were selected on the basis of their fast growing habit and with different colony characteristics. Isolates were examined for their plant growth promoting activities such as nitrogen fixation^20^, phosphate solubilization^21^ and production of indole acetic acid^22^, cellulase^23^ and siderophores^24^. Sixteen positive isolates showing at least one functional activity were selected for present study (Table 1). The selected bacterial strains were characterized by 16S rRNA gene (rDNA) sequence analysis as done earlier^20^. These strains were assembled in two groups. Ten phylogenetically diverse nitrogen fixing isolates were used for constructing communities of nitrogen-fixing bacteria (hereafter called as nitrogen-fixing bacteria). Twelve phylogenetically diverse but possessing different functional activities (six nitrogen fixers, two phosphate solubilizers, two indole acetic acid producers, one cellulose producer and one siderophore producer) were considered for constituting communities in case of phylogenetically as well as functionally diverse communities of rhizospheric bacteria (hereafter called as rhizospheric bacteria; Table S1a and S1b in Supplementary Information).

### Experimental design and treatment**s**

One-month-old seedlings obtained from germinating surface sterilized seeds under sterile conditions were taken for study. The potting material consisted of soil: sand (2:1) mixture sieved through 2.5 mm mesh and then autoclaved for 3 h at 121 °C in three consecutive cycles within 48 hours. The soil was sandy loam (Ustifluvent) with pH, 7.19; electrical conductivity, 0.41 dS m^−1^; organic carbon, 4.47 g kg^−1^; available N (alkaline permanganate extractable), 123 kg ha^−1^; available P (0.46 M NaHCO_3_ extractable), 9.8 kg ha^−1^; and available K (1 N NH_4_OAc extractable), 95 kg ha^−1^.

Bacterial species were assembled in various combinations with different diversity gradients (1, 2, 3, 4, 6, 12 for rhizospheric bacteria and 1, 2, 5, 10 for nitrogen-fixing bacteria) through random samplings^17^. At all richness levels, species combinations were made randomly without replacement such that each species is selected equally at each of the richness level (Supplementary Information Tables S2 and S3). The process of constructing a set of experimental units was carried out independently three times at each richness level, and each of the combination was replicated three times. In addition, un-inoculated control with three replications (without any bacterial inoculation) was also included.

Bacterial cultures were prepared by growing them in 10 ml nutrient broth for 24 h in an orbital incubator shaker (200 rpm at 28 °C), harvesting cells by centrifuging at 8000 × g for 5 min at 4 °C and diluting their pellets with sterile saline water (0.85%) to a CFU (colony forming units) of ∼10^8^ mL^−1^. CFU was determined by plating serially diluted 0.1 ml culture on nutrient agar plates after incubating for 24 hours. For preparing inoculum of different combinations, an equal quantity of each bacterial culture was mixed in a sterile vial so that total individual density in each combination remains similar. Roots of one-month-old seedlings were dipped into bacterial culture for one hour and transplanted into the pots (20 cm high and 20 cm internal diameter). Additionally, 2 ml of culture (10^8^ CFU mL^−1^) containing appropriate bacterial combinations in equal amounts was placed into the planting hole. To maintain uniformity, control plants were inoculated with autoclaved saline water. The pots were kept in a glass house and were irrigated with sterilized water as and when required.

### Measurements of plant biomass, soil microbial respiration

Plants were harvested 60 days after transplanting (at the initiation of flowering), and total biomass of oven dried plant material was recorded. Microbial respiration was estimated from the potting soil sieved through 2 mm mesh after manually removing plant residues. A sample of 50 g soil was kept in airtight glass jars with 1N NaOH in a separate tube. Jars were incubated for 10 days at 28 °C. CO_2_ evolution by microbial respiration was estimated through titrating the NaOH with 1N HCl and expressed as g CO_2_ kg^−1^ day^−1^, based on dry weight^25^.

### Statistical analyses

The relationship between response variables (plant biomass, soil microbial respiration) and predicting variables (species richness, functional group richness) was analyzed through linear regression. Pairwise differences between individual species and between different levels of species richness were determined with one-way ANOVA with Tukey’s post hoc test. In a particular composition, if monocultures didn’t differ in performance, transgressive overyielding was not calculated and was reported as zero. When differences among monocultures were significant at P < 0.05 level, transgressive overyielding was calculated as the difference between mixture yield and yield of most performing species present in the mixture.


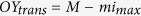


where *OY*_*trans*_ is transgressive overyielding and *mi*_*max*_ is the maximum monoculture yield.

The effects of species richness (linear and fixed factor), species identity and compositions were analysed with general linear models in R version 3.1.0 (http://www.r-project.org) following Bell *et al.*^26^. The advantage of the separation of species richness into linear and fixed factor is that once linear richness has been entered into the model, the effect of species identity and the effect of nonlinear species richness are orthogonal (i.e., do not share sums of squares). In this analysis, the nonlinear richness explains the effect of species interaction on the response variable. For avoiding the problem of pseudoreplication, F-statistic was calculated with specific error terms. The effects of species richness were tested against partitioned species pools and the effects of species identities against compositions. Diagnostic plots of residuals versus fitted values revealed the lack of significant heterogeneity of variance and Q-Q plots showed that assumptions of normality were justified. Cook’s distances were tested to examine the level of influence of extreme data points. No data point was noticed influential to change the output of analyses and interpretation of results.

## Results

**Figure 1:**
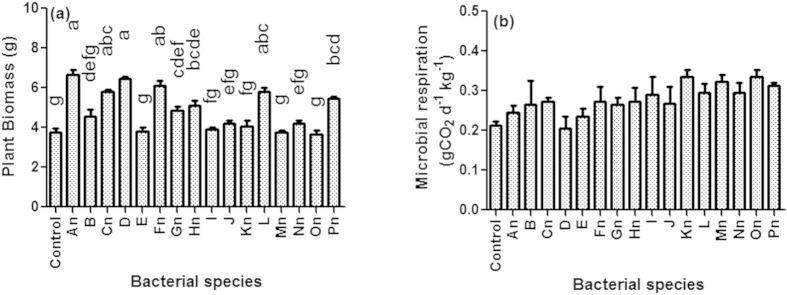
Effect of bacterial species monocultures, inoculated in the rhizosphere of *Ocimum sanctum*, on plant biomass (**a**) and soil microbial respiration (**b**). Bars followed by different letters were significantly different at P < 0.05 level (Tukey’s test). Error bars represent ± 1SEM. ndenotes the strains which were included in nitrogen fixing bacterial group.

Plant biomass varied on inoculations with different bacteria in monocultures (Fig. 1a). Although all the bacterial species were found to express plant growth promoting activity *in vitro*, only 50% of them significantly increased the plant biomass as compared to control. Two bacteria i.e. *Klebsiella pneumoniae* and *Brevibacillus agri* were found to be better than others in increasing plant growth in monoculture. On the other hand, 60% of nitrogen-fixing bacteria were found to be plant growth promoters in pot experiments (Fig. 1a). Soil microbial respiration was not significantly different among species monocultures, and it was also not different from control suggesting the species individually couldn’t increase the microbial respiration over control as analysed through our protocol (Fig. 1b).

**Figure 2:**
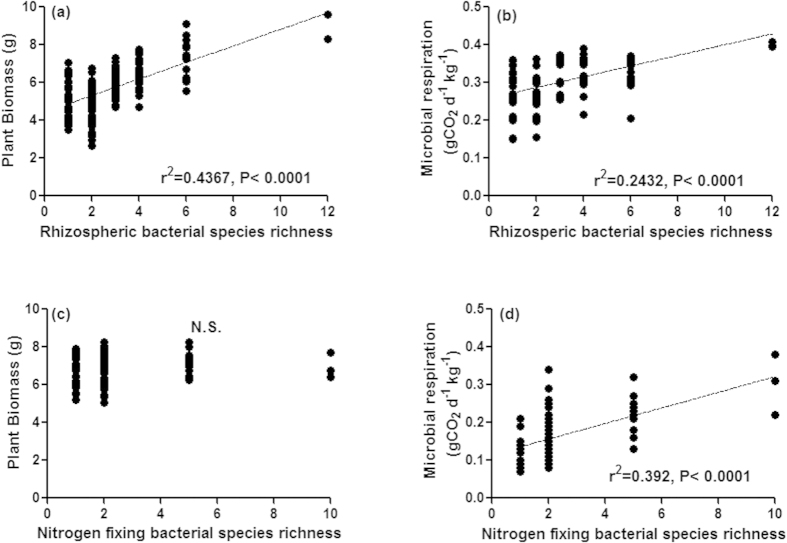
Effect of bacterial communities varying in species richness, inoculated in the rhizosphere of *Ocimum sanctum*, on plant biomass and soil microbial respiration. Effect of rhizospheric bacterial communities varying from one to twelve species richness on plant biomass (**a**) and soil microbial respiration (**b**). Effect of nitrogen-fixing bacterial communities varying from one to ten species richness on plant biomass (**c**) and soil microbial respiration (**d**).

Plant biomass increased linearly with inoculation of increasing diversity of rhizospheric bacterial diversity (Fig. 2; F_1, 172_ = 133.4, P < 0.0001), on the other hand, no relationship was found between plant biomass and nitrogen-fixing bacterial species richness (F_1, 94_ = 1.055, P = 0.3071). Species richness, however, greatly increased soil microbial respiration (F_1, 172_ = 5.27, P < 0.0001 for rhizospheric bacteria and F_1, 94_ = 60.51, P < 0.0001 for nitrogen-fixing bacteria). Interestingly, some species-poor compositions performed similar to some compositions of higher richness levels indicating the positive effects of the presence of particular species in the compositions in both the cases.

**Figure 3:**
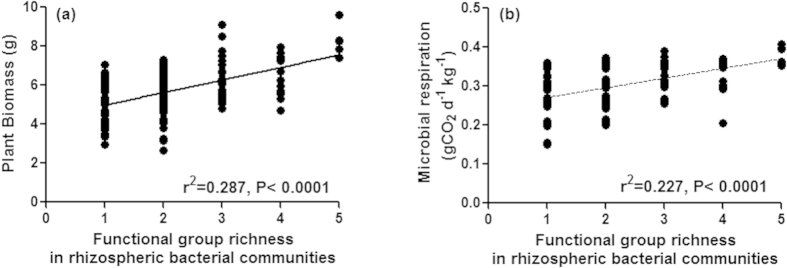
Effect of functional group richness in rhizospheric bacterial communities, inoculated in the rhizosphere of *Ocimum sanctum*, on plant biomass (**a**) and soil microbial respiration (**b**).

As species richness was found to be an important predictor of improved plant biomass and respiration, we tried to establish if the functional diversity of plant growth promoting traits within species richness further enhances the performance of a community. A strong positive relationship was also observed between functional group richness and dependent variables (plant biomass and soil microbial respiration, Fig. 3). In our experiments, though species richness and functional group richness were observed to be important but found to be highly correlated (r = 0.84), and therefore it is difficult to establish their exclusive effects.

**Figure 4:**
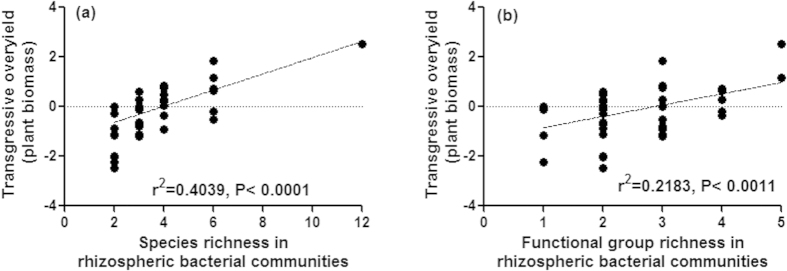
Transgressive over yield in plant biomass at different levels of species richness (**a**) and functional group richness (**b**) in rhizospheric bacterial communities inoculated in the rhizosphere of *Ocimum sanctum*.

The presence of transgressive overyielding indicates the positive biodiversity effects may be due to niche complementation or mutualistic interactions. Transgressive overyielding was calculated only for plant biomass in case of rhizospheric bacteria where monocultures differed in their performance. At lower levels of species richness transgressive underyielding was also observed, however, at higher levels overyielding was prevalent. A significant relationship between transgressive overyielding and species richness was observed (Fig. 4; F_1, 44_ = 29.81, P < 0.0001). A similar relationship was observed between transgressive overyielding and functional group richness. As monocultures within nitrogen-fixing bacterial group did not differ among themselves in their ability to promote plant growth and microbial respiration in the soil, no such analyses were performed in this case.

In linear model analyses, the linear effect of species richness on plant biomass and respiration was significant. However, in case of nitrogen-fixing bacteria the relation between species richness and plant biomass (Tables 2 and 3) was not noticed. The significant linear relationship between species richness and ecosystem functioning indicates complementary effects of biodiversity where the presence of any new species has some additional increase in ecosystem functioning. The linear effect of species richness was significant in the case of rhizospheric bacteria only, and the absence of this effect in the case of nitrogen-fixing bacterial group shows the redundancy of functional traits in this group. Fixed effects of species richness on plant biomass were not significant at all, in any case. The fixed effect shows the effect of species richness above the complementarity, where the deviation from linearity indicates the strength of species interactions. Interestingly, the effects of species identities were found significant in the case of soil respiration in nitrogen-fixing bacteria. The effect of particular species compositions on plant biomass and soil microbial respiration was consistently significant in all the cases. It shows that regardless of the general pattern of diversity-functioning relationship, specific compositions of some species, at all richness levels, have a large effect.

## Discussion

Functional diversity is assumed a strong predictor of ecosystem functioning^27^ because it can approximate niche heterogeneity in a community. We found that functionally diverse rhizospheric bacterial communities enhance plant productivity while functionally redundant nitrogen fixing community did not; the plant biomass could not increase over monocultures. Linear increase in ecosystem functioning and presence of transgressive overyielding in several combinations indicate that bacterial functional traits may work in a complementary way and enhance plant biomass. These results fall in the line of several earlier reports, where increasing bacterial richness increased ecosystem functioning of the system^8^,^9^,^17^. However, in these reports as well as in ours, some species combinations at lower richness levels were also observed to be as productive as the higher richness levels, indicating the effects of species identities in these compositions. We found significant antagonistic interactions at lower levels of species richness in case of rhizospheric bacteria, however, these were infrequent and non-significant at higher levels of species richness. It might be due to the modification of species interactions such as direct competition in the presence of other species. An increase in soil respiration with species richness in case of both experiments indicates that bacterial diversity positively affects the functioning of the microbial community, however, its impact on plant growth depends on diverse plant growth promoting activities.

Diversity effects are overall outcomes of several types of negative and positive interactions such as facilitation and competition in microbial communities. Bacterial strains, which lack a particular activity when tested alone, may act synergistically in promoting ecosystem functioning when included as a part of microbial consortia^5^. These interactions were reflected in transgressive underyielding in case of plant biomass, where the yield of plants inoculated with species mixture was significantly lower than maximum yielding monocultures. Similar negative biodiversity- ecosystem functioning relationships were obtained by Becker *et al.*^13^. Besides direct antagonism among species such as toxin production^28^, competition for root exudates and space^29^ may also play a significant role in underyielding in plant biomass. Accumulation and/or dominance of underproductive species may also exert considerable influence in underyielding. The overall effect of diversity is affected by species interactions in which the positive and negative effects may neutralize each other, and the net diversity effect may be zero^30^. However, at higher richness levels, this deleterious effect may be compensated by the presence of many highly productive species indicating the sampling effect of biodiversity^31^. Our observations indicate that role of bacterial diversity in the rhizosphere ranges from positive to negative interactions that may also counteract each other at some instances. It is also found that plant actively recruits beneficial microorganisms in rhizosphere probably for increasing its fitness^5^ through antagonising harmful microorganisms. The probability of including beneficial microorganisms increases with diversity compensating the deleterious effects caused by opportunists. It is supported by our results also where increasing trends in diversity effects (overyielding) with species richness were observed. At higher levels of richness, the beneficial effects superimpose deleterious one and overall improvement in ecosystem services may be achieved.

Several limitations should be considered before generalising the results of present and all similar type of studies. The diversity of our system was very low, and the observations are based on short-term response to initial diversity. The competitive relationships among species may, however, change with long-term observations due to the extirpation of some species. In the present experiment, only a minor part of the variation in plant growth could be explained by bacterial richness, however, this would remain an active question of research that may provide better understanding of rhizosphere ecosystem functioning. In conclusion, we have shown that complementarity among different plant growth promoting traits within the community is useful in increasing plant productivity. These observations would be helpful in understanding plant-microbe interactions in the rhizosphere.

## Additional Information

**How to cite this article**: Singh, M. *et al.* Complementarity among plant growth promoting traits in rhizospheric bacterial communities promotes plant growth. *Sci. Rep.* **5**, 15500; doi: 10.1038/srep15500 (2015).

## Supplementary Material

Supplementary Information

## Figures and Tables

**Table 1:** Bacterial species used in the study, as identified through 16 s rRNA gene sequence analysis.

Sp. Id	Closest NCBI match	*NCBI Accession number	Functional activity
A_n_	*Klebsiella pneumoniae*	JX512025	Nitrogen fixer
B	*Staphylococcus pasteuri*	JX512027	IAA Producer
C_n_	*Bacillus cereus*	JX512029	Nitrogen fixer
*D*	*Brevibacillus agri*	JX512031	Phosphate solubilizer
E	*Bacillus subtilis*	JX512032	Phosphate solubilizer
F_n_	*Pseudomonas putida*	JX512033	Nitrogen fixer
G_n_	*Bacillus megaterium*	JX512035	Nitrogen fixer
H_n_	*Enterobacter sp.*	JX512036	Nitrogen fixer
I	*Cronobacter sakazakii*	JX512037	Siderophore producer
J	*Pantoea agglomerans*	JX512038	Cellulase producer
K_n_	*Alcaligenes sp.*	JX512039	Nitrogen fixer
L	*Micrococcus sp.*	JX512040	IAA Producer
M_n_	*Bacillus thuringiensis*	JX512028	Nitrogen fixer
N_n_	*Bacillus firmus*	JX512034	Nitrogen fixer
O_n_	*Pseudomonas rhizosphaerae*	JX512030	Nitrogen fixer
P_n_	*Flexibacter sp.*	JX512026	Nitrogen fixer

Sequences have been submitted to NCBI GenBank. Please refer to Supplementary Information for separate list of species used in two experiments.

_n_ denotes the strains which were included in nitrogen fixing bacterial group.

**Table 2:** Results of general linear models showing the effects of species richness, species identities and compositions of rhizospheric bacterial communities inoculated in the rhizposphere of *Ocimum sanctum* on plant biomass and microbial respiration.

Factors	df	Plant biomass			Microbial respiration		
		SS	F	P	SS	F	P
Sp. Richness							
Linear	1, 12	120.3	42.09	2.99 × 10^−4^	0.1263	8.982	0.0111
Fixed	6, 8	14.59	0.99	0.492	0.07988	1.2	0.394
Sp. ID	12, 46	14.47	0.60	0.8339	0.01973	0.438	0.9389
Compositions	58, 116	93.08	5.84	4.14 × 10^−16^	0.17248	2.846	8.5 × 10^−7^

Sp. Richness, species richness; Sp. ID, species identity; df, degrees of freedom; SS, sum of squares; F, F-statistic; P, P-value.

**Table 3:** Results of general linear models showing the effects of species richness, species identities and compositions of nitrogen fixing bacterial communities inoculated in the rhizposphere of *Ocimum sanctum* on plant biomass and microbial respiration.

Factors	df	Plant biomass			Microbial respiration		
		SS	F	P	SS	F	P
Sp. Richness							
Linear	1, 6	0.552	0.487	0.511	0.153	60.508	9.27 × 10^−12^
Fixed	4, 4	1.003	0.173	0.941	0.005	0.4945	0.7398
Sp. ID	10, 22	2.712	0.296	0.975	0.023	0.94	0.51
Compositions	32, 64	20.118	1.586	0.058	0.110	2.196	0.004

Sp. Richness, species richness; Sp. ID, species identity; df, degrees of freedom; SS, sum of squares; F, F-statistic; P, P-value.
