# Supplementary material for: Complementarity among plant growth promoting traits in rhizospheric bacterial communities promotes plant growth
Source: Sci Rep. 2015 Oct 27;5:15500. doi: 10.1038/srep15500 (PMC4621411; doi:10.1038/srep15500)
Supplement: Supplementary Information [file srep15500-s1.pdf]

Complementarity among plant growth promoting traits in rhizospheric bacterial communities promotes plant growth

Mangal Singh, Ashutosh Awasthi, Sumit K. Soni, Rakshapal Singh, Rajesh K. Verma, Alok Kalra*

Department of Microbial Technology and Entomology, CSIR- Central Institute of Medicinal and Aromatic Plants, P.O. CIMAP, Lucknow 226015, India

Table S1a: List of 12 bacterial species used in rhizospheric bacterial group

Sp. Id	Closest NCBI match	*NCBI Accession number	Functional activity
A_n	*Klebsiella pneumoniae*	JX512025	Nitrogen fixer
B	*Staphylococcus pasteuri*	JX512027	IAA Producer
C_n	*Bacillus cereus*	JX512029	Nitrogen fixer
D	*Brevibacillus agri*	JX512031	Phosphate solubilizer
E	*Bacillus subtilis*	JX512032	Phosphate solubilizer
F_n	*Peudomonas putida*	JX512033	Nitrogen fixer
G_n	*Bacillus megaterium*	JX512035	Nitrogen fixer
H_n	*Enterobacter sp.*	JX512036	Nitrogen fixer
I	*Cronobacter sakazakii*	JX512037	Siderophore producers
J	*Pantoea agglomerans*	JX512038	Cellulase producer
K_n	*Alcaligenes sp.*	JX512039	Nitrogen fixer
L	*Micrococcus sp.*	JX512040	IAA Producer

Table S1b: List of 10 bacterial species used in nitrogen fixing bacterial group

Sp. Id	Closest NCBI match	*NCBI Accession number	Functional activity
A_n	*Klebsiella pneumoniae*	JX512025	Nitrogen fixer
C_n	*Bacillus cereus*	JX512029	Nitrogen fixer
F_n	*Peudomonas putida*	JX512033	Nitrogen fixer
G_n	*Bacillus megaterium*	JX512035	Nitrogen fixer
H_n	*Enterobacter sp.*	JX512036	Nitrogen fixer
K_n	*Alcaligenes sp.*	JX512039	Nitrogen fixer
M_n	*Bacillus thuringiensis*	JX512028	Nitrogen fixer
N_n	*Bacillus firmus*	JX512034	Nitrogen fixer
O_n	*Pseudomonas rhizosphaerae*	JX512030	Nitrogen fixer
P_n	*Flexibacter sp.*	JX512026	Nitrogen fixer

Table S2: Details of experimental design and species compositions of the rhizospheric bacterial group

Composition	Richness	An	B	C_n	D	E	F_n	G_n	H_n	I	J	K_n	L
1	1	1	0	0	0	0	0	0	0	0	0	0	0
2	1	1	0	0	0	0	0	0	0	0	0	0	0
3	1	1	0	0	0	0	0	0	0	0	0	0	0
4	1	0	1	0	0	0	0	0	0	0	0	0	0
5	1	0	1	0	0	0	0	0	0	0	0	0	0
6	1	0	1	0	0	0	0	0	0	0	0	0	0
7	1	0	0	1	0	0	0	0	0	0	0	0	0
8	1	0	0	1	0	0	0	0	0	0	0	0	0
9	1	0	0	1	0	0	0	0	0	0	0	0	0
10	1	0	0	0	1	0	0	0	0	0	0	0	0
11	1	0	0	0	1	0	0	0	0	0	0	0	0
12	1	0	0	0	1	0	0	0	0	0	0	0	0
13	1	0	0	0	0	1	0	0	0	0	0	0	0

14	1	0	0	0	0	1	0	0	0	0	0	0	0
15	1	0	0	0	0	1	0	0	0	0	0	0	0
16	1	0	0	0	0	0	1	0	0	0	0	0	0
17	1	0	0	0	0	0	1	0	0	0	0	0	0
18	1	0	0	0	0	0	1	0	0	0	0	0	0
19	1	0	0	0	0	0	0	1	0	0	0	0	0
20	1	0	0	0	0	0	0	1	0	0	0	0	0
21	1	0	0	0	0	0	0	1	0	0	0	0	0
22	1	0	0	0	0	0	0	0	1	0	0	0	0
23	1	0	0	0	0	0	0	0	1	0	0	0	0
24	1	0	0	0	0	0	0	0	1	0	0	0	0
25	1	0	0	0	0	0	0	0	0	1	0	0	0
26	1	0	0	0	0	0	0	0	0	1	0	0	0
27	1	0	0	0	0	0	0	0	0	1	0	0	0
28	1	0	0	0	0	0	0	0	0	0	1	0	0
29	1	0	0	0	0	0	0	0	0	0	1	0	0
30	1	0	0	0	0	0	0	0	0	0	1	0	0
31	1	0	0	0	0	0	0	0	0	0	0	1	0
32	1	0	0	0	0	0	0	0	0	0	0	1	0
33	1	0	0	0	0	0	0	0	0	0	0	1	0
34	1	0	0	0	0	0	0	0	0	0	0	0	1
35	1	0	0	0	0	0	0	0	0	0	0	0	1
36	1	0	0	0	0	0	0	0	0	0	0	0	1
37	2	1	1	0	0	0	0	0	0	0	0	0	0
38	2	1	1	0	0	0	0	0	0	0	0	0	0
39	2	1	1	0	0	0	0	0	0	0	0	0	0
40	2	0	0	1	1	0	0	0	0	0	0	0	0
41	2	0	0	1	1	0	0	0	0	0	0	0	0
42	2	0	0	1	1	0	0	0	0	0	0	0	0
43	2	0	0	0	0	1	1	0	0	0	0	0	0
44	2	0	0	0	0	1	1	0	0	0	0	0	0
45	2	0	0	0	0	1	1	0	0	0	0	0	0
46	2	0	0	0	0	0	0	1	1	0	0	0	0
47	2	0	0	0	0	0	0	1	1	0	0	0	0
48	2	0	0	0	0	0	0	1	1	0	0	0	0
49	2	0	0	0	0	0	0	0	0	1	1	0	0
50	2	0	0	0	0	0	0	0	0	1	1	0	0
51	2	0	0	0	0	0	0	0	0	1	1	0	0
52	2	0	0	0	0	0	0	0	0	0	0	1	1
53	2	0	0	0	0	0	0	0	0	0	0	1	1
54	2	0	0	0	0	0	0	0	0	0	0	0	1
55	2	1	0	1	0	0	0	0	0	0	0	0	0
56	2	1	0	1	0	0	0	0	0	0	0	0	0
57	2	1	0	1	0	0	0	0	0	0	0	0	0
58	2	0	0	0	1	0	1	0	0	0	0	0	0

59	2	0	0	0	1	0	1	0	0	0	0	0	0
60	2	0	0	0	1	0	1	0	0	0	0	0	0
61	2	0	0	0	0	0	0	1	0	1	0	0	0
62	2	0	0	0	0	0	0	1	0	1	0	0	0
63	2	0	0	0	0	0	0	1	0	1	0	0	0
64	2	0	1	0	0	0	0	0	0	0	1	0	0
65	2	0	1	0	0	0	0	0	0	0	1	0	0
66	2	0	1	0	0	0	0	0	0	0	1	0	0
67	2	0	0	0	0	1	0	0	0	0	0	0	1
68	2	0	0	0	0	1	0	0	0	0	0	0	1
69	2	0	0	0	0	1	0	0	0	0	0	0	1
70	2	0	0	0	0	0	0	0	1	0	0	1	0
71	2	0	0	0	0	0	0	0	1	0	0	1	0
72	2	0	0	0	0	0	0	0	1	0	0	1	0
73	2	1	0	0	1	0	0	0	0	0	0	0	0
74	2	1	0	0	1	0	0	0	0	0	0	0	0
75	2	1	0	0	1	0	0	0	0	0	0	0	0
76	2	0	0	1	0	0	0	0	0	0	1	0	0
77	2	0	0	1	0	0	0	0	0	0	1	0	0
78	2	0	0	1	0	0	0	0	0	0	1	0	0
79	2	0	0	0	0	0	1	0	0	0	0	1	0
80	2	0	0	0	0	0	1	0	0	0	0	1	0
81	2	0	0	0	0	0	1	0	0	0	0	1	0
82	2	0	1	0	0	0	0	1	0	0	0	0	0
83	2	0	1	0	0	0	0	1	0	0	0	0	0
84	2	0	1	0	0	0	0	1	0	0	0	0	0
85	2	0	0	0	0	1	0	0	0	1	0	0	0
86	2	0	0	0	0	1	0	0	0	1	0	0	0
87	2	0	0	0	0	1	0	0	0	1	0	0	0
88	2	0	0	0	0	0	0	0	1	0	0	0	1
89	2	0	0	0	0	0	0	0	1	0	0	0	1
90	2	0	0	0	0	0	0	0	1	0	0	0	1
91	3	1	1	1	0	0	0	0	0	0	0	0	0
92	3	1	1	1	0	0	0	0	0	0	0	0	0
93	3	1	1	1	0	0	0	0	0	0	0	0	0
94	3	0	0	0	1	1	1	0	0	0	0	0	0
95	3	0	0	0	1	1	1	0	0	0	0	0	0
96	3	0	0	0	1	1	1	0	0	0	0	0	0
97	3	0	0	0	0	0	0	1	1	1	0	0	0
98	3	0	0	0	0	0	0	1	1	1	0	0	0
99	3	0	0	0	0	0	0	1	1	1	0	0	0
100	3	0	0	0	0	0	0	0	0	0	1	1	1
101	3	0	0	0	0	0	0	0	0	0	1	1	1
102	3	0	0	0	0	0	0	0	0	0	1	1	1
103	3	1	0	0	1	0	1	0	0	0	0	0	0

104	3	1	0	0	1	0	1	0	0	0	0	0	0
105	3	1	0	0	1	0	1	0	0	0	0	0	0
106	3	0	1	0	0	1	0	0	0	1	0	0	0
107	3	0	1	0	0	1	0	0	0	1	0	0	0
108	3	0	1	0	0	1	0	0	0	1	0	0	0
109	3	0	0	1	0	0	0	0	1	0	0	0	1
110	3	0	0	1	0	0	0	0	1	0	0	0	1
111	3	0	0	1	0	0	0	0	1	0	0	0	1
112	3	0	0	1	0	0	0	1	0	0	1	0	0
113	3	0	0	1	0	0	0	1	0	0	1	0	0
114	3	0	0	1	0	0	0	1	0	0	1	0	0
115	3	1	0	0	0	1	0	0	1	0	0	0	0
116	3	1	0	0	0	1	0	0	1	0	0	0	0
117	3	1	0	0	0	1	0	0	1	0	0	0	0
118	3	0	1	0	0	0	1	0	0	1	0	0	0
119	3	0	1	0	0	0	1	0	0	1	0	0	0
120	3	0	1	0	0	0	1	0	0	1	0	0	0
121	3	0	0	1	0	0	0	1	0	0	0	1	0
122	3	0	0	1	0	0	0	1	0	0	0	1	0
123	3	0	0	1	0	0	0	1	0	0	0	1	0
124	3	0	0	0	1	0	0	0	0	0	1	0	1
125	3	0	0	0	1	0	0	0	0	0	1	0	1
126	3	0	0	0	1	0	0	0	0	0	1	0	1
127	4	1	1	1	1	0	0	0	0	0	0	0	0
128	4	1	1	1	1	0	0	0	0	0	0	0	0
129	4	1	1	1	1	0	0	0	0	0	0	0	0
130	4	0	0	0	0	1	1	1	1	0	0	0	0
131	4	0	0	0	0	1	1	1	1	0	0	0	0
132	4	0	0	0	0	1	1	1	1	0	0	0	0
133	4	0	0	0	0	0	0	0	0	1	1	1	1
134	4	0	0	0	0	0	0	0	0	1	1	1	1
135	4	0	0	0	0	0	0	0	0	1	1	1	1
136	4	1	0	0	0	1	0	0	0	1	0	0	1
137	4	1	0	0	0	1	0	0	0	1	0	0	1
138	4	1	0	0	0	1	0	0	0	1	0	0	1
139	4	0	1	0	0	0	1	0	1	0	1	0	0
140	4	0	1	0	0	0	1	0	1	0	1	0	0
141	4	0	1	0	0	0	1	0	1	0	1	0	0
142	4	0	0	1	1	0	0	1	0	0	0	1	0
143	4	0	0	1	1	0	0	1	0	0	0	1	0
144	4	0	0	1	1	0	0	1	0	0	0	1	0
145	4	1	0	1	0	0	1	0	0	1	0	0	0
146	4	1	0	1	0	0	1	0	0	1	0	0	0
147	4	1	0	1	0	0	1	0	0	1	0	0	0
148	4	0	1	0	1	0	0	1	0	0	0	1	0

149	4	0	1	0	1	0	0	1	0	0	0	1	0
150	4	0	1	0	1	0	0	1	0	0	0	1	0
151	4	0	0	0	0	1	0	0	1	0	1	0	1
152	4	0	0	0	0	1	0	0	1	0	1	0	1
153	4	0	0	0	0	1	0	0	1	0	1	0	1
154	6	1	1	1	1	1	1	0	0	0	0	0	0
155	6	1	1	1	1	1	1	0	0	0	0	0	0
156	6	1	1	1	1	1	1	0	0	0	0	0	0
157	6	0	0	0	0	0	0	1	1	1	1	1	1
158	6	0	0	0	0	0	0	1	1	1	1	1	1
159	6	0	0	0	0	0	0	1	1	1	1	1	1
160	6	1	0	1	0	1	0	0	0	1	1	0	1
161	6	1	0	1	0	1	0	0	0	1	1	0	1
162	6	1	0	1	0	1	0	0	0	1	1	0	1
163	6	0	1	0	1	0	1	1	1	0	0	1	0
164	6	0	1	0	1	0	1	1	1	0	0	1	0
165	6	0	1	0	1	0	1	1	1	0	0	1	0
166	6	0	1	0	1	0	1	1	0	1	0	1	0
167	6	0	1	0	1	0	1	1	0	1	0	1	0
168	6	0	1	0	1	0	1	1	0	1	0	1	0
169	6	1	0	1	0	1	0	0	1	0	1	0	1
170	6	1	0	1	0	1	0	0	1	0	1	0	1
171	6	1	0	1	0	1	0	0	1	0	1	0	1
172	12	1	1	1	1	1	1	1	1	1	1	1	1
173	12	1	1	1	1	1	1	1	1	1	1	1	1
174	12	1	1	1	1	1	1	1	1	1	1	1	1

Table S3: Details of experimental design and species compositions of the nitrogen fixing bacterial group

Composition	Richness	A_n	C_n	F_n	G_n	H_n	K_n	M_n	N_n	O_n	P_n
1	1	1	0	0	0	0	0	0	0	0	0
2	1	1	0	0	0	0	0	0	0	0	0
3	1	1	0	0	0	0	0	0	0	0	0
4	1	0	1	0	0	0	0	0	0	0	0
5	1	0	1	0	0	0	0	0	0	0	0
6	1	0	1	0	0	0	0	0	0	0	0
7	1	0	0	1	0	0	0	0	0	0	0
8	1	0	0	1	0	0	0	0	0	0	0
9	1	0	0	1	0	0	0	0	0	0	0

10	1	0	0	0	1	0	0	0	0	0	0
11	1	0	0	0	1	0	0	0	0	0	0
12	1	0	0	0	1	0	0	0	0	0	0
13	1	0	0	0	0	1	0	0	0	0	0
14	1	0	0	0	0	1	0	0	0	0	0
15	1	0	0	0	0	1	0	0	0	0	0
16	1	0	0	0	0	0	1	0	0	0	0
17	1	0	0	0	0	0	1	0	0	0	0
18	1	0	0	0	0	0	1	0	0	0	0
19	1	0	0	0	0	0	0	1	0	0	0
20	1	0	0	0	0	0	0	1	0	0	0
21	1	0	0	0	0	0	0	1	0	0	0
22	1	0	0	0	0	0	0	0	1	0	0
23	1	0	0	0	0	0	0	0	1	0	0
24	1	0	0	0	0	0	0	0	1	0	0
25	1	0	0	0	0	0	0	0	0	1	0
26	1	0	0	0	0	0	0	0	0	1	0
27	1	0	0	0	0	0	0	0	0	1	0
28	1	0	0	0	0	0	0	0	0	0	1
29	1	0	0	0	0	0	0	0	0	0	1
30	1	0	0	0	0	0	0	0	0	0	1
31	2	1	1	0	0	0	0	0	0	0	0
32	2	1	1	0	0	0	0	0	0	0	0
33	2	1	1	0	0	0	0	0	0	0	0
34	2	0	0	1	1	0	0	0	0	0	0
35	2	0	0	1	1	0	0	0	0	0	0
36	2	0	0	1	1	0	0	0	0	0	0
37	2	0	0	0	0	1	1	0	0	0	0
38	2	0	0	0	0	1	1	0	0	0	0
39	2	0	0	0	0	1	1	0	0	0	0
40	2	0	0	0	0	0	0	1	1	0	0
41	2	0	0	0	0	0	0	1	1	0	0
42	2	0	0	0	0	0	0	1	1	0	0
43	2	0	0	0	0	0	0	0	0	1	1
44	2	0	0	0	0	0	0	0	0	1	1
45	2	0	0	0	0	0	0	0	0	1	1
46	2	1	0	1	0	0	0	0	0	0	0
47	2	1	0	1	0	0	0	0	0	0	0
48	2	1	0	1	0	0	0	0	0	0	0
49	2	0	0	0	1	0	1	0	0	0	0
50	2	0	0	0	1	0	1	0	0	0	0
51	2	0	0	0	1	0	1	0	0	0	0
52	2	0	0	0	0	0	0	1	0	1	0
53	2	0	0	0	0	0	0	1	0	1	0
54	2	0	0	0	0	0	0	1	0	1	0

55	2	0	1	0	0	0	0	0	0	0	1
56	2	0	1	0	0	0	0	0	0	0	1
57	2	0	1	0	0	0	0	0	0	0	1
58	2	0	0	0	0	1	0	0	1	0	0
59	2	0	0	0	0	1	0	0	1	0	0
60	2	0	0	0	0	1	0	0	1	0	0
61	2	1	0	0	1	0	0	0	0	0	0
62	2	1	0	0	1	0	0	0	0	0	0
63	2	1	0	0	1	0	0	0	0	0	0
64	2	0	0	1	0	0	0	0	0	0	1
65	2	0	0	1	0	0	0	0	0	0	1
66	2	0	0	1	0	0	0	0	0	0	1
67	2	0	0	0	0	0	1	0	1	0	0
68	2	0	0	0	0	0	1	0	1	0	0
69	2	0	0	0	0	0	1	0	1	0	0
70	2	0	1	0	0	0	0	1	0	0	0
71	2	0	1	0	0	0	0	1	0	0	0
72	2	0	1		0	0	0	1	0	0	0
73	2	0	0	0	0	1	0	0	0	1	0
74	2	0	0	0	0	1	0	0	0	1	0
75	2	0	0		0	1	0	0	0	1	0
76	5	1	1	1	1	1	0	0	0	0	0
77	5	1	1	1	1	1	0	0	0	0	0
78	5	1	1	1	1	1	0	0	0	0	0
79	5	0	0	0	0	0	1	1	1	1	1
80	5	0	0	0	0	0	1	1	1	1	1
81	5	0	0	0	0	0	1	1	1	1	1
82	5	1	0	1	0	1	0	1	0	1	0
83	5	1	0	1	0	1	0	1	0	1	0
84	5	1	0	1	0	1	0	1	0	1	0
85	5	0	1	0	1	0	1	0	1	0	1
86	5	0	1	0	1	0	1	0	1	0	1
87	5	0	1	0	1	0	1	0	1	0	1
88	5	1	0	0	1	0	1	0	1	1	0
89	5	1	0	0	1	0	1	0	1	1	0
90	5	1	0	0	1	0	1	0	1	1	0
91	5	0	1	1	0	1	0	1	0	0	1
92	5	0	1	1	0	1	0	1	0	0	1
93	5	0	1	1	0	1	0	1	0	0	1
94	10	1	1	1	1	1	1	1	1	1	1
95	10	1	1	1	1	1	1	1	1	1	1
96	10	1	1	1	1	1	1	1	1	1	1
